# Case report: Imaging findings of true thymic hyperplasia at ^18^F-FDG PET/CT in an infant

**DOI:** 10.3389/fonc.2022.1077777

**Published:** 2023-01-06

**Authors:** Jiazhong Ren, Zheng Fu, Yaqing Zhao

**Affiliations:** ^1^ Department of Medical Imaging, PET-CT Center, Shandong Cancer Hospital and Institute, Shandong First Medical University and Shandong Academy of Medical Sciences, Jinan, Shandong, China; ^2^ Department of General Affairs Section, The Second Affiliated Hospital of Shandong University of Traditional Chinese Medicine, Jinan, Shandong, China

**Keywords:** ^18^F‐FDG PET/CT, CT, mediastinum, TTH, MTH

## Abstract

True thymic hyperplasia (TTH) in children is rare and difficult to distinguish from other thymic tumors such as thymoma and thymic carcinoma. A 3‐year‐old girl underwent an ^18^F‐fluorodeoxyglucose (^18^F‐FDG) positron emission tomography (PET)/computed tomography (CT) scan (^18^F‐FDG PET/CT) and a chest CT scan to evaluate an anterior mediastinal mass. ^18^F‐FDG PET/CT revealed a mediastinal mass showing heterogeneously increased FDG uptake with a maximum standardized uptake value (SUVmax) of 7.1. Eventually, postoperative pathological diagnosis demonstrated TTH. So far, there are no reports of ^18^F‐FDG PET/CT imaging of this disease.

## Introduction

True thymic hyperplasia (TTH) usually presents as an anterior mediastinal mass and causes significant challenges in its diagnosis and treatment (1). The enlargement of the thymus occurs most often during infancy and usually spontaneously degenerates after the age of 3 years (2). TTH is the most common benign tumor observed in pediatric patients with tumors following chemotherapy and is essentially a rebound of the thymus gland (3). However, TTH in children is rare, mostly has unknown etiology, and is usually not with any comorbidity (4). Computed tomography (CT) and magnetic resonance imaging (MRI) are mainly used to evaluate thymic lesions. ^18^F‐fluorodeoxyglucose positron emission tomography/CT (^18^F‐FDG PET/CT) has an excellent diagnostic value in thymic rebound and can differentiate between thymic hyperplasia (TH), thymoma, and thymic carcinoma (5). However, there are fewer reports on the diagnostic value of ^18^F‐FDG PET/CT in scanning children with TTH. Here, we describe the case of a child whose diagnosis of TTH was based on a pathological diagnosis after surgical resection, which was supported by CT‐enhanced imaging and an ^18^F‐FDG PET/CT scan. ^18^F‐FDG PET/CT is of great value in the preoperative evaluation of pediatric patients with TTH.

## Case presentation

**Figure 1:**
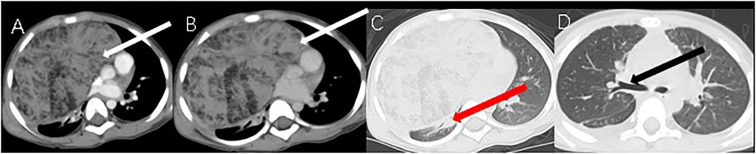
CT images.

A 3‐year‐old girl was admitted to the hospital owing to a 20‐day history of cough and sputum without hemoptysis. Physical examination revealed an anterior chest bulge measuring approximately 6 × 5 cm^2^, firm in texture, with poor mobility, and with no sternal pressure and rubbing sensation in the chest. She had no other symptoms and no family history of TTH or other diseases. Laboratory tests showed a mildly elevated lactate dehydrogenase (LDH) at 276 U/L (reference range: 38–126 U/L), a white blood cell count of 13.76 × 10^9^/L (reference range: 4–10 × 10^9^/L), and a red blood cell count of 5.09 × 10^12^/L (reference range: 4–4.5 × 10^12^/L). However, thyroid‐related laboratory test results were normal. In addition, neuron‐specific enolase (NSE) and human chorionic gonadotropin levels (HCG) were normal. The chest CT scan revealed a well‐defined irregular mass of 17 × 10 × 7.5 cm^3^ in the right anterior mediastinum, with strip‐like low‐density shadows in the lesion, and a CT value of approximately −67 Hounsfield units, thereby suggesting adipose tissue (**Figure 1**). The arterial phase showed less vascularity and heterogeneous hypodensity within the lesion (A, white arrow; **Figure 1**); the venous phase showed persistent hypodensity (B, white arrow; **Figure 1**) with compression of the cardiac cavity, right main bronchus, and occlusion of the right main bronchus (C, red arrow; **Figure 1**) ; the compressed right main bronchus and corresponding lung tissue returned to normal (D, black arrow; **Figure 1**) one week after the patient underwent chest surgery.

**Figure 2:**
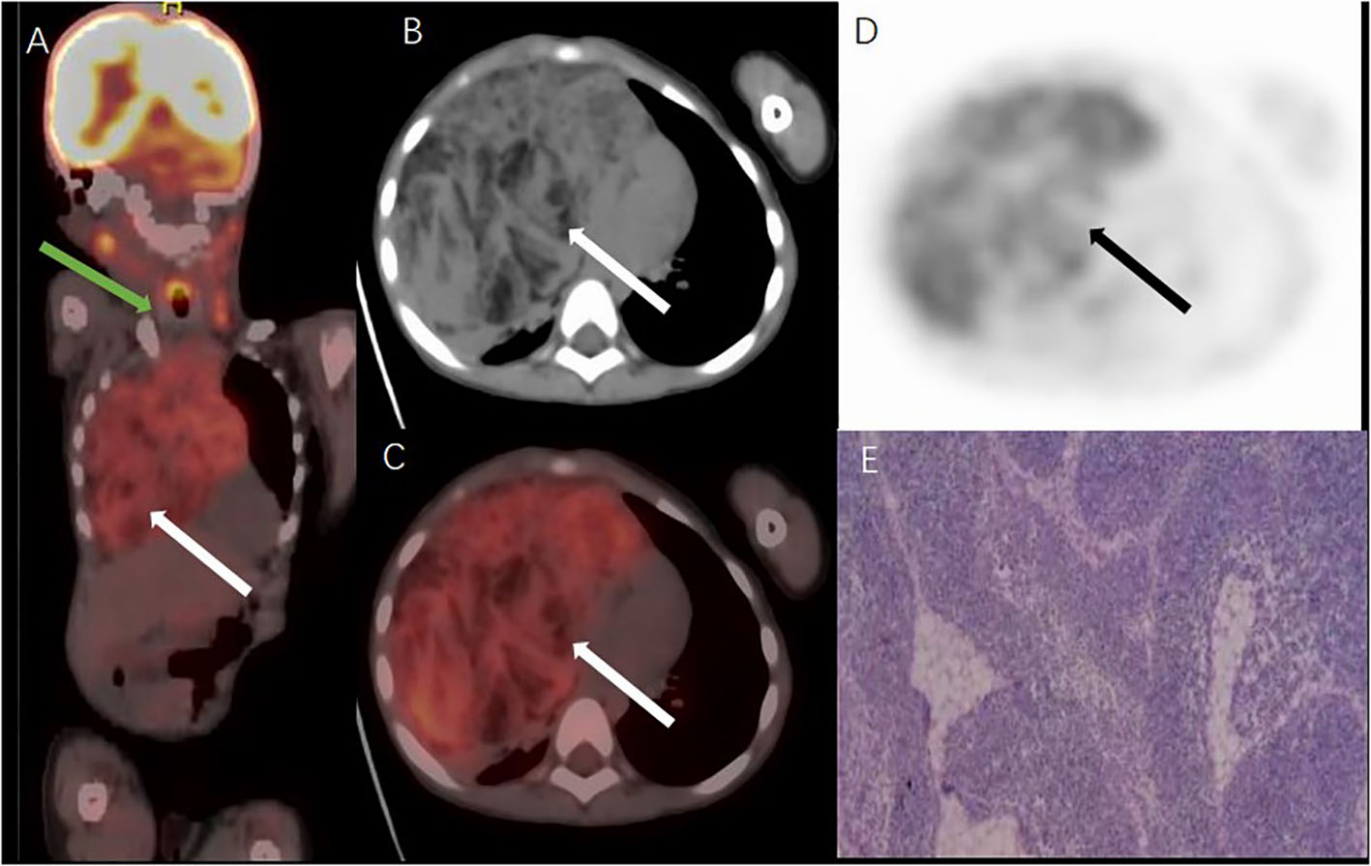
18F FDG PETCT images and pathologic picture.

After a CT scan of the chest, ^18^F‐FDG PET/CT was performed owing to concerns that the lesion may be malignant. Coronal PET/CT fusion images (A; **Figure 2**) show a large, heterogeneous hypermetabolic mass in the right thorax (white arrows; **Figure 2**), whereas the thyroid was normal and not hypermetabolic (green arrows; **Figure 2**), thereby suggesting a thymic origin of the mass. In axial images (B: CT; C: PET/CT fusion; D: PET; **Figure 2**), the uptake of FDG with an SUVmax of 7.1 (white and black arrows; **Figure 2**) shows that the mass has heterogeneous hyperactivity. Pathological images (E, hematoxylin–eosin stain; original magnification × 100) showed histologically normal thymic tissue consisting of lobules with well‐defined cortical and medullary cells. Immunohistochemical staining was positive for CKpan, CK19, LCA, CD3, TDT, MDM2, and CD1a and negative for CK20, and Desmin. The lesion was eventually diagnosed as TTH, and the patient recovered well during the follow‐up period.

## Discussion

The thymus is a gland situated in the anterior mediastinum; it is embryologically derived from the pharyngeal pouch of the third and fourth branchial arch (2). Its size varies with age because thymic tissue gradually shrinks and is replaced by adipose tissues. The thymus gland gradually invaginates during the first 3–4 years of life (6).

TH is usually divided into two categories: TTH and lymphatic follicular hyperplasia (LFH) (7). TTH is characterized by a diffused thickening and enlargement of the thymus that exceeds the corresponding upper limit of size or mass in a normal child of the same age but maintains the normal thymic structure and immunohistochemical features (8). TH is often caused by chemotherapy for tumors, thermal burns, or surgery. LFH is characterized by an increase in the number of lymphoid follicles and germinal centers in the thymus, which may be normal or slightly enlarged in size. LFH is associated with several endocrine and autoimmune diseases such as Graves’ disease, myasthenia gravis, and systemic lupus erythematosus (9).

TTH in children is a rare clinical condition, has unknown etiology, and often has no comorbidities. It is often referred to as massive thymic hyperplasia (MTH) because it is characterized by a relatively larger and heavier thymus than the thymus of a healthy individual. The diagnostic features of MTH are as follows: 1. radiographs show gland projection exceeding the cardiac shadow, 2. The thymus weighs several times its expected weight at a specific age, 3. the mass of the thymus is > 2% of the body mass, and 4. pathological test shows normal thymus structure (10). The patient in this case report largely met the above diagnostic criteria.

The most common tumors of the anterior mediastinum in children are tumors of thymic, lymphatic, or germ‐cell origin (11). Tumor markers (alpha‐fetoprotein (AFP), HCG, and NSE) are helpful in the differential diagnosis of some tumors such as germ cell tumors (GCTs). Mediastinal yolk sac tumor is often associated with elevated levels of serum AFP, seminoma with elevated levels of serum HCG, and lymphoma with elevated levels of LDH. MRI or CT scans are often used to evaluate thymic lesions (11, 12). Mediastinal GCTs are mostly teratomas, often containing fatty tissues and calcifications, and are easily diagnosed. Invasion of the thymus by lymphoma usually occurs in the setting of extensive systemic disease. Homogeneous enlargement of the thymus with mediastinal and/or axillary lymph node enlargement is usually diagnosed as lymphoma (13). Previous studies have reported that ^18^F‐FDG PET/CT can differentiate between benign and malignant thymic tumors but cannot distinguish between aggressive and non‐aggressive thymoma and still relies on morphological examination by CT and MRI or pathological examination (8, 14, 15). In general, benign uptake of physiological thymus or chemotherapy‐induced thymic rebound hyperplasia is less intense on ^18^F‐FDG PET/CT scans, where SUVmax is approximately 1.0–2.8. In comparison to thymoma and TH, thymic carcinoma shows considerably higher FDG uptake (13).

Although multimodal imaging has an excellent diagnostic value for TTH, MRI can more easily identify mediastinal masses, which are directly contiguous with and follow the same signal features as the main body of the thymus (12). Moreover, MRI is more sensitive in detecting adipose tissue in TTH, which can enable us to accurately deduce the origin and nature of the mass. Therefore, we recommend that MRI should always be preferred in diagnosing pediatric patients if feasible, specifically because it is free of ionizing radiation and is therefore safe for these patients. Unfortunately, due to our inexperience with TTH and the suspicion that the mediastinal mass was malignant as detected by enhanced CT, we selected ^18^F‐FDG PET/CT instead of MRI for further examination.

In this case report, ^18^F‐FDG PET/CT scan revealed that there was an increase in the heterogeneous hypermetabolism at the lesion with an SUVmax of 7.1, the lung tissue adjacent to the lesion was only compressed but not invaded, and the patient had no lymph node metastases and distant metastases. Although ^18^F‐FDG PET/CT has limited diagnostic value for thymic lesions (8, 12), especially in children, in this case report, it helped us to determine that the lesion was benign with no adjacent tissue invasion, which enabled subsequent surgery plans for the patient.

Most previous case reports suggest that treatment of TTH with steroids is usually ineffective; therefore, surgical resection is a better option (6, 10, 16). Finally, the patient underwent complete surgical resection of the mediastinal tumor and recovered well after surgery without any postoperative complications. Therefore, we recommend that surgical resection should be the first option for pediatric patients with TTH because this tumor is non‐invasive to adjacent tissues and easily resectable, and the surgery is free of any postoperative complications.

In conclusion, ^18^F‐FDG PET/CT is of great value in the preoperative diagnosis and assessment of pediatric patients with TTH. In addition, awareness of these findings is important in the interpretation of PET/CT scans of anterior mediastinal masses in young children.

## Data availability statement

The original contributions presented in the study are included in the article/supplementary material. Further inquiries can be directed to the corresponding author.

## Ethics statement

The studies involving human participants were reviewed and approved by Ethics Committee of Cancer Hospital Affiliated to Shandong First Medical University. Written informed consent to participate in this study was provided by the participants’ legal guardian/next of kin. Written informed consent was obtained from the minor(s)’ legal guardian/next of kin for the publication of any potentially identifiable images or data included in this article.

## Author contributions

JR acquisition of data, drafting of the manuscript; ZF and YZ: revision of the manuscript, supervision. All authors read and critically revised the manuscript for intellectual content and approved the final manuscript.
